# Improving genomic prediction of vitamin C content in spinach using GWAS-derived markers

**DOI:** 10.1186/s12864-025-11343-0

**Published:** 2025-02-21

**Authors:** Jana Jeevan Rameneni, A S M Faridul Islam, Carlos A. Avila, Ainong Shi

**Affiliations:** 1 Texas A&M AgriLife Research and Extension Center, 2415 Highway 83, Weslaco, TX 78596 USA; 2 Department of Horticultural Sciences, Texas A&M University, College Station, TX 77843 USA; 3 Vegetable and Fruit Improvement Center, Texas A&M University, College Station, TX 77845 USA; 4 Department of Horticulture, 316 Plant Sciences Building (PTSC), University of Arkansas, Fayetteville, AR 72701 USA

**Keywords:** Genomic Selection, Genome-Wide Association Study, Spinach, SNP, Prediction Accuracy

## Abstract

Vitamin C (VC), also known as ascorbic acid and ascorbate, is a water-soluble antioxidant in plants that promotes skin health and immune function in humans. Spinach (*Spinacia oleracea* L.) is a leafy green widely consumed for its health benefits. Recent reports have shown that nutritional content, including VC, can be improved in spinach. However, due to its complex inheritance, new selection methods are needed to improve selection for cultivar development. In this study, single nucleotide polymorphism (SNP) markers identified by genome-wide association (GWAS) were used for genomic prediction (GP) to improve prediction accuracy (PA) for VC content in spinach. A set of 147,977 SNPs generated from whole genome resequencing was used for GWAS in a panel of 347 spinach genotypes by six GWAS models. Sixty-two SNP markers distributed on all six spinach chromosomes were associated with VC content. PA for the selection of VC content was estimated with fourteen random SNP sets across seven GP models. The results indicated that the PA can be > 40% after using 1,000 or more SNPs in six of the seven models tested; using GWAS-derived significant SNP markers PA increases to a high *r*-value up to 0.7 when using 62 associated SNP markers in Bayes ridge regression (BRR) model. Upon validation, identified accessions with high VC and high PA genomic selection model can be used in spinach breeding programs to develop high VC content cultivars.

**Supplementary Information:**

The online version contains supplementary material available at 10.1186/s12864-025-11343-0.

## Introduction

Cultivated spinach (*Spinacia oleracea* L.) is a cool-season, dark green leafy vegetable consumed worldwide [1]. The global market for spinach is valued at $39.6 billion, with approximately 31 million tons produced worldwide. Most of the spinach production occurs in Asia (95.2%), with China being the top producer with 28 million tons [2, 3]. The United States is the second-largest spinach producer, with 367,433 tons cultivated across 56,200 acres in 2020 [2, 3]. Spinach consumption has steadily increased at a rate of 4.6% annually from 2012 to 2020 due to its rich nutritional profile [3]. L-ascorbic acid or ascorbate, commonly known as Vitamin C (VC), is an essential antioxidant compound found in spinach leaves, as well as many minerals, other vitamins, fibers, and phenolic compounds such as flavonoids and polyphenols.

VC has many biological functions in both plants and humans, but while plants can synthetize it, humans have to meet the VC requirements from the diet (90% of VC is obtained from fruits and vegetables) [4]. As an antioxidant molecule, its primary function in humans is to neutralize reactive oxygen species (ROS), along with reducing oxidative stress-related conditions, such as cancer, cardiovascular diseases, and aging [5]. Within plants, VC serves as both an antioxidant and enzymatic cofactor, involved in numerous physiological processes, including growth and development, photosynthesis, photoprotection, stress resistance, regulation of cell growth, and the synthesis of hormones and cell wall components [5]. Moreover, a diverse range of environmental and developmental changes, such as light and temperature, diurnal and seasonal fluctuations, aging, plant tissue and cell compartment, and abiotic stresses like drought and salinity affects ascorbic acid content in plants [5, 6]. Several reports indicate that increasing VC in plants results in increased biomass and enhanced abiotic stress tolerance (e.g. salinity, heat, UV, etc.) [5, 7, 8].

Despite the prominent functions of VC in plants and humans, limited research has been reported to enhance its content in spinach due to its complex inheritance for its use in conventional breeding programs [6]. While in other crops or plant models such as Arabidopsis, lettuce, potato, tomato, tobacco, and maize, genetic engineering techniques have shown success in improving VC content [5, 7, 8], genetic engineering is not a current viable approach in spinach improvement due to consumers concerns about genetically modified crops. Therefore, tools to improve VC selection in breeding programs are needed to enhance VC content using conventional approaches.

Genome-wide association studies (GWAS) are useful to identify loci associated with target traits [9–11]. GWAS analysis has been widely employed in different agricultural and horticultural crops, including tomato [12–16], eggplant [17, 18], and potato [19–21] for the prediction of genetic loci (SNP’s/QTLs/ genes) associated with important agricultural traits. In spinach, Cai et al. [22] identified loci associated with leaf and flower-related traits using GWAS. Similarly, various studies applied this approach in spinach for the identification of genetic loci associated with leaf traits [23], mineral content [24], oxalate concentration [25], leaf miner resistance (*Liriomyza* spp.) [26], Verticillium wilt resistance [27], Stemphylium leaf spot resistance [28], downy mildew resistance [29], and white rust resistance [30] using diverse spinach accessions.

In addition to GWAS, genomic selection (GS) has become a vital tool in crop improvement programs. In comparison to marker-assisted selection, which is limited to specific major genes or alleles, GS can select many loci simultaneously and account for infinitesimally small effect alleles [31, 32]. GS estimates marker effect genome-wide, and is used to calculate the genomic estimated breeding values (GEBV) [33]. In brief, both phenotyping and genotyping for a trait of interest are conducted in a training population, followed by developing a statistical or machine learning model based on the training population that can measure the genome-estimated breeding value of individuals in a breeding population [32]. So far, various types of estimation models have been used to calculate GEBV, which can be categorized into five methods: (1) stepwise regression, (2) ridge regression, (3) Bayesian estimation models like Bayes A, Bayes B, Bayes LASSO, (4) semi-parametric regressions like kernel-based regression and neural networks, and (5) machine learning methods like random forest and support vector machine models [33]. GS has many advantages, including reducing the need for phenotyping, increasing selection intensity, and enabling selection based on alleles rather than line means [34, 35]. This approach has been successfully applied in a variety of crops such as rice, maize, soybean, wheat, and common bean for identifying the SNPs associated to agronomical, biotic and abiotic stress-related traits [35–45].

Unfortunately, GWAS approach has constraints such as limited predictive value, genetic risk, population demographics, uncovering all source of genomic variants for complex traits [46]. Utilization of GWAS coupled with GS can improve the prediction accuracy, and also overcome limitations of GWAS. Earlier reports and research studies on combing these two approaches shown increased prediction accuracy for qualitative traits in different crop species [47–49].

Due to its importance as an antioxidant in plants and health-promoting functions in humans, VC is a highly desirable trait for improvement in spinach breeding to improve abiotic stress tolerance and enhance post-harvest shelf-life quality. However, due to its complex inheritance, breeding tools need to be developed to efficiently and quickly select plants with high content. In this study, we aimed to improve prediction accuracy (PA) by using GWAS- identified single nucleotide polymorphism (SNP) markers in genomic prediction (GP) models with a panel of 347 spinach accessions. Estimations of PA were evaluated for different marker subsets to identify the best combination for VC content selection in spinach.

## Materials and methods

### Plant materials

A total of 347 spinach accessions were used to conduct GWAS and GP analyses for VC content. This GWAS panel has been used in previous studies and reports [50, 51] Out of the total, 337 accessions were obtained from USDA-NPGS, originally collected from 30 countries. The rest of the evaluated accessions included ten spinach breeding lines obtained from Pop Vriend Seeds (Table S1).

### Phenotyping and data analysis

The experiment was conducted under growth chamber at an average temperature of 23 °C, 11 h light, and a light intensity of 120 μmol/m^2^/s in a complete randomized design (CRD) with 3-replications. Leaf tissues were collected from 2-month-old plants into 15 ml tubes containing steal metal beads, flash frozen, and stored in −80 °C for further analysis. During the whole process, samples were kept on ice and away from direct light to prevent ascorbic acid oxidation. Leaf tissues were homogenized for 35 s at 1500 rpm and added 2.25 ml of ice-cold 6% metaphosphoric acid and mixed thoroughly. Further, 0.75 ml of leaf extract was transferred to new 2 ml tubes in duplicates and centrifuged at 14000 rpm for 5 min at 4 °C, then the supernatant was transferred to new 1.5 ml tubes. The absorbance of supernatant was further measured in spectrophotometer at 265 nm, and VC concentration was estimated as described earlier studies [50–52]. The VC content data among the 347 spinach accessions were analyzed using the analysis of variance (ANOVA) with the general linear models (GLM) procedure of JMP Genomics 7 (SAS Institute, Cary, NC). The mean, range, standard deviation (SD), standard error (SE), and coefficient of variation (CV) were estimated for VC using ‘Tabulate’ function. The distribution of VC was drawn using ‘Distribution’ in JMP Genomics 7. The average VC content for each spinach accession from ANOVA was used as the phenotypic data for GWAS. The broad sense heritability (H^2^) was estimated using the following formula [53].

$$\text{H}2 =\upsigma 2\text{g }/ [\upsigma 2\text{g }+ (\upsigma 2\text{e }/\text{ r})]$$

with σ^2^_g_ being the total genetic variance, σ^2^_e_ being the residual variance, and r is the number of replications. The estimates for σ^2^_g_ and σ^2^_e_ were [EMS(G)- Var(Residual)]/ r and Var(Residual), respectively. EMS(G) and Var(Residual) were obtained from the ANOVA table. Where EMS (G) was the expected mean squares of genotype and Var(Residual) refers to the variation in data that is not explained by the ANOVA model.

### Genotyping

DNA was extracted from fresh leaves pooled from 5–10 plants per genotype. Before sequencing, high-quality DNA of each sample was randomly shared into 350-bp fragments by Covaris Ultrasonic Processor. The construction of the DNA libraries followed the process of end repairing, adding A tails, purification, PCR amplification, and library qualification. The DNA libraries were paired end sequenced using whole-genome resequencing (WGR) technology on Illumina NovaSeq platform, achieving 10X coverage of the spinach genome and generating approximately 10 Gb of sequencing data per sample at BGI (https://www.bgi.com/). The spinach genome of Monoe-Viroflay genome annotation [22](http://spinachbase.org/ftp/genome/Monoe-Viroflay/) and Sp75 [54, 55](available at SpinachBase) were used as references to map the WGR data of the 347 spinach genotypes using Burrows-Wheeler aligner software (BWA v0.7.8-r455). SAMtools (v 0.1.19–44428 cd) were utilized to sort the bam files and remove duplication reads. The program Picard (v 1.111) [56] was used to merge the bam files from the same sample, and the GATK software (v 3.5) [57] was chosen to detect and filter SNPs and InDels. Around 16 million raw SNPs were identified in the 347 spinach genotypes. Filtering and keeping the SNPs with minor allele frequency (MAF) > 2%, missing allele < 30%, and heterogeneous rate < 50%, retained 147,977 high-quality SNPs based on Monoe-Viroflay genome reference and were used in this study (Fig. S1). All genomic sequences generated and used for the spinach accessions in this study have been deposited at the National Center for Biotechnology Information (NCBI) Sequence Read Archive (SRA) under BioProject ID: PRJNA860974.

### Principal component analysis (PCA), population structure and genetic diversity

In this study, 147,977 SNPs were used for population structure, principal component analysis (PCA) and phylogenetic analyses (Fig. S2). PCA and phylogenetic analyses were conducted using GAPIT 3 software (Genomic Association and Prediction Integrated Tool version 3) [58] (https://zzlab.net/GAPIT/index.html; https://github.com/jiabowang/GAPIT3) by setting PCA = 2 to 10 and phylogenetic tree = 2 to 10. SNP diversity in the phylogenetic tree was analyzed using the neighbor-joining (NJ) method. Population structure was performed with admixture model using STRUCTURE version v.2.3.4 software [6, 59].

### Association analysis

GWAS was performed using the 147,977 SNPs across the 347 spinach genotypes by SMR (single marker regression), GLM and MLM (mixed linear model) models in TASSEL 5 software [60] and MLM, FarmCPU (fixed and random model circulating probability unification), and BLINK (bayesian-information and linkage-disequilibrium iteratively nested keyway) models in GAPIT 3 software [58] by setting PCA = 3. Additionally, a *t*-test was performed on all SNPs using visual basic codes in Microsoft Excel 2016.

Multiple models of TASSEL and GAPIT tools were used to find reliable and stable SNP markers and candidate genes associated to VC content in the GWAS panel. The significant threshold of associations was calculated using Bonferroni correction of *P*-value with an α = 0.05 (0.05 / SNP number) as the significance threshold [61], and LOD [-log(*P*-value); we use LOD instead in this article] value of 6.47 was used as significance threshold based on the 147,977 SNPs in this study.

### Candidate gene identification/detection

Linkage disequilibrium analysis was performed to predict the haplotype blocks using Plink software v.2.0 [62] with the parameters as described in previous study [63]. Candidate genes were searched within 50 Kb on either side of significant SNPs of the spinach Monoe-Viroflay genome annotation at the FTP site (http://spinachbase.org/ftp/genome/Monoe-Viroflay/) and SpinachBase (http://www.spinachbase.org/). Our emphasis was to find genes near the significantly associated SNP markers.

### Genomic prediction for genomic selection of vitamin C content

GP was performed using various statistical machine learning models, including Bayesian models Bayes A (BA), Bayes B (BB), Bayes LASSO (BL), and Bayes ridge regression (BRR), of BGLR package [64], Ridge regression best linear unbiased prediction (rrBLUP) of rrBLUP package [65], Random forests (RF), and Support vector machines (SVM) of kernlab packages R [66]. VC- associated SNPs predicted by the models in the whole genome were cross- validated using a five-fold ratio (4:1 of training / testing sets). Eighty percent of genotypes were assigned randomly into 14 subsets (r12, r62, r200, r500, r1000, r2000, r5000, r10000, r15000, r20000, r30000, r40000, r50000, and r60000) and used as a training set and the remaining 20% of genotypes were assigned to 2 subsets, (GWAS-derived SNP markers, m12, and m62) and treated as test set, collectively makes 16 sets that were distributed on seven GP models. The PA for the tested models in this study was estimated by calculating the average Pearson’s correlation coefficient (r) between the GEBVs estimates from the training set and VC content phenotypic values in the validation set or testing set (Zhang et al. 2016; Shi et al. 2021, 2022; Shikha et al. 2017). Average *r*-values generated from training and testing sets were used in the analysis to construct the distribution charts.

## Results

### Vitamin C content phenotyping

**Fig. 1:**
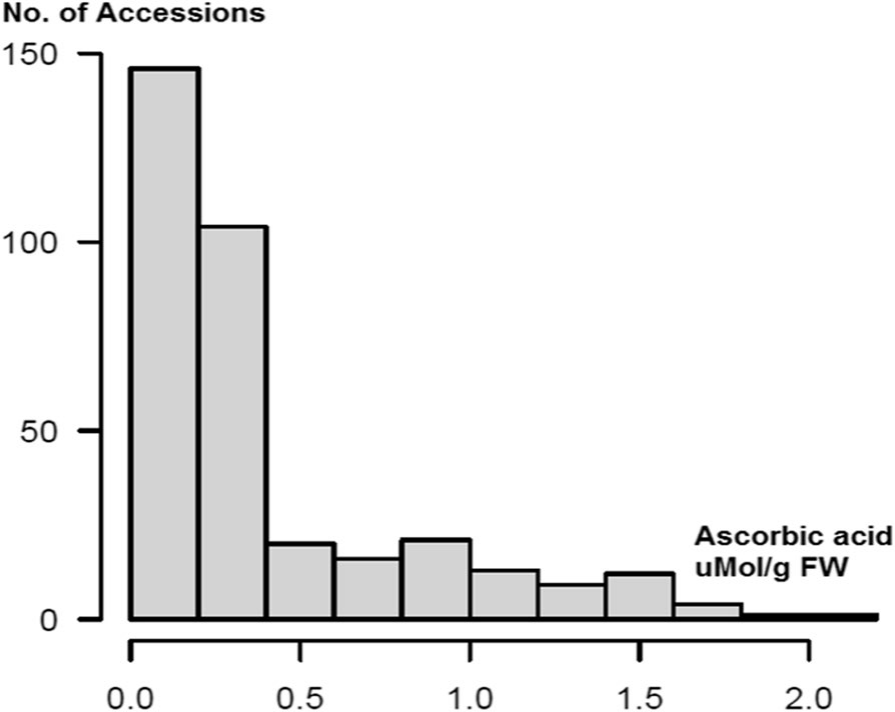
The distribution of ascorbic acid (AsA) content in 347 spinach lines. The y-axis denotes number of accessions and the x-axis denotes the concentration of VC across the leaf samples germplasm

The VC content in the 347-spinach genotypes shows a skewed distribution with most of the accessions having low VC content in the panel (Fig. 1, Table S1). The mean VC content ranged between 0.036 to 2.122 (µMol/g FW = fresh weight). The panel had an average of 0.406 (µMol/g FW) with a standard deviation of 0.415, and the CV is 102.2%. The lowest VC content accession was PI 604783 with 0.036 (µMol/g FW) and the highest PI 648940 with 2.122 (µMol/g FW) representing a ~ 60X fold between them, indicating extensive range and variation of the VC content in the 347 spinach genotypes (Table S1), confirming the suitability of this panel for GWAS analyses.

Twelve out of the 347 spinach genotypes showed high VC content > 1.5 (µMol/g FW) including five USDA GRIN spinach accessions and seven breeding lines from Pop Vriend Seeds (Table 1), which can be used in spinach breeding as parents for improving VC content.

**Table 1:** Top 12 spinach lines with highest ascorbic acid (AsA) content. The table shows the line accession IDs given by respective organization (USDA, and Pop Vriend Seeds), country of origin, phylogeny cluster and VC content

ACCESSION	Country	2-cluster	AsA content (µMol/g FW)
PI 648940	China	G2	2.122
PI 648948	China	G2	1.556
PI 664498	China	G1	1.832
PI 173129	Turkey	G1	1.547
PI 648958	United States	G1	1.589
PV1515	Pop Vriend Seeds	G1	1.695
PV1517	Pop Vriend Seeds	G1	1.562
PV_1444	Pop Vriend Seeds	G1	1.696
PV_1445	Pop Vriend Seeds	G1	1.743
PV_1446	Pop Vriend Seeds	G1	1.718
PV_1452	Pop Vriend Seeds	G1	1.509
PV_1477	Pop Vriend Seeds	G1	1.532

Mean squares for the spinach genotype was significant (*P* ≤ 0.001) for the VC content (Table S2). The broad sense heritability (H^2^) was 87.4% for VC content (Table S2), indicating the VC content was highly inheritable.

### Genetic diversity analysis using structure, PCA and phylogenetic analysis

The genotype stratification of the panel of 347 accessions was estimated using respective genotypic data in STRUCTURE software with K values 1 to 10. The results of the analysis show that the accessions can be grouped into 3 true clusters based on the K value on the X-axis as shown in Fig. S2A, this indicates that these spinach accessions majorly have 3 sub-populations. As seen in the probability cut-off value SQ = 0.5, the accessions can be classified into SQ1, and SQ2 and whereas SQ3 is genetic admixture of SQ2 and SQ1 sub-populations (Fig. S2A).

**Fig. 2:**
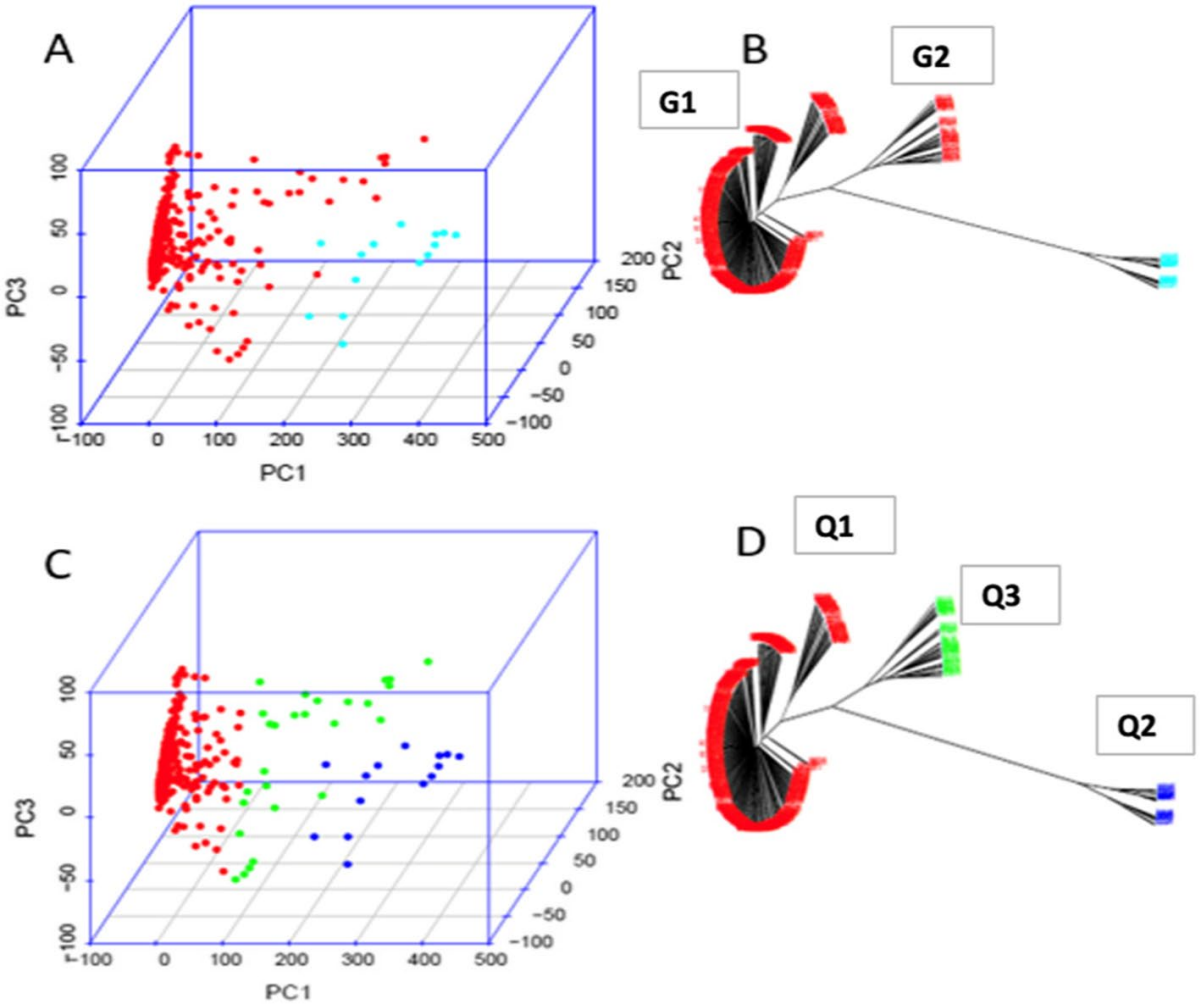
Population structure and genetic diversity analysis of 347 spinach lines where PCA = 2 (**A** and **B**) and PCA = 3 (**C** and **D**). A and C represents 3D graphical plots of the principal component analysis (PCA), and **B** and **D** represents unrooted phylogenetic tree of the genotypes constructed using neighbor-joining (NJ) method of GAPIT 3 software

**Fig. 3:**
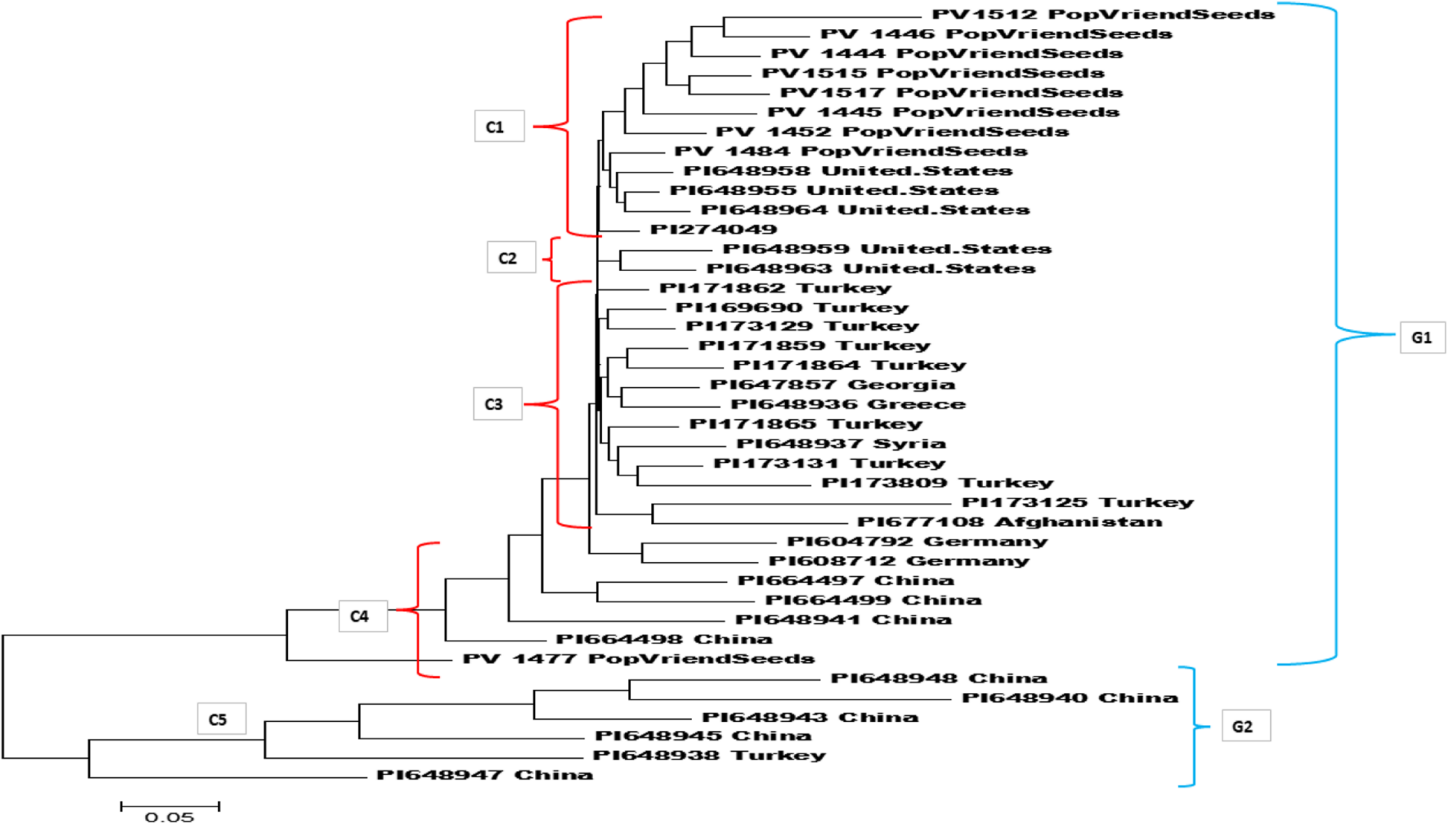
Phylogenetic tree of thirty-one USDA GRIN Accessions and nine lines from Pop Vriend Seeds with high ascorbic acid (AsA) content. G1,G2 and C1-C4 indicates group and cluster classification

Similarly, principal component analysis (PCA) was conducted with GAPIT 3 software by setting PCA parameters ranging from 2 to 10 to investigate the population genetic structure among 347 spinach accessions. The analysis identified two distinct clusters/groups, designated as G1 and G2, at PCA = 2. Group1 was comprised of a mixture of different regions, ranging from Western Asia to Europe, Africa, North America, and even a small proportion of South Asian accessions. In contrast, Group-2 predominantly contained East and South Asian spinach accessions, with a small proportion of accessions from Western Asia (Turkey) (Fig. 3: Fig. S2B). At PCA = 3, the second cluster (G2) was observed to be further subdivided into two sub-clusters, resulting in a total of three distinct clusters (designated as Q1, Q2, and Q3) for 347 spinach accessions (Fig. 2). A genotype-based phylogenetic analysis using the neighbor-joining (NJ) method was also performed in GAPIT 3 and the analysis reflected similar clustering pattern of principal component analysis (PCA), demonstrating three sub-clusters in the evaluated spinach germplasm (Fig. 2; Fig. S2).

Among 347 spinach accessions, 88% (306 accessions) and 8% (27 accessions) of spinach germplasm were grouped into the first sub-cluster (Q1) and third sub-cluster (Q3), respectively; the Q2 sub-cluster was composed of only 14 accessions (4% of spinach germplasm). Except in the Q2 sub-cluster, accessions of unknown origin and Pop Vriend Seeds were clustered into Q1 and Q3 (Fig. 2; Fig.S2).

In terms of accession’s proportion based on accession’s origin in each cluster, Q1 sub-cluster consisted of accessions from seven regions (East Africa, Eastern Asia, Europe, North Africa, North America, Southern Asia, and Western Asia), with eight accessions from Pop Vriend Seeds and 18 accessions of unknown origin. The highest proportion of accessions (40% accessions of Q1) came from western Asia countries (Iran, Iraq, Syria, and Turkey), followed by Europe (22%) and North America (18%) region. The remaining two sub-clusters (Q2 and Q3) were composed of accessions from three regions (Eastern Asia, Southern Asia, and Western Asia) including 2 Pop Vriend Seeds accession in Q3, with different proportions. Interestingly, 11 out of 14 accessions in Q2 were from Eastern Asia (79%) countries, followed by 14% accessions of Southern Asia countries. In contrast, half of Q3 cluster’s accessions (52%) came from Southern Asia countries and 37% of accessions were from Eastern Asia regions. In both Q2 and Q3 clusters, Western Asia’s accessions were the lowest (7% in Q2 and 4% in Q3) (Fig. 2; Fig.S2).

With respect to the proportion of accessions across the clusters per region, all accessions from East and North Africa, Europe, and North America along with 98% of Western Asia’s accessions were gathered into the first cluster (Q1). More than half (57%) of Southern Asia’s accessions were grouped into the Q1 cluster, followed by 38% of accessions of Southern Asia were joined into the third cluster (Q3). Among four southern Asia countries, 20 of 22 Afghanistan’s accessions were grouped into Q1, but 92% of Indian accessions (11 of 12 accessions) were clustered into Q3. Accessions of Pakistan and Nepal were joined into Q2 cluster along with the rest of the accessions of Afghanistan and India. For the Eastern Asia region, 30 accessions were distributed in almost equal proportion into three clusters. China’s 22 accessions were almost equally divided between Q1 (8 accessions) and Q2 (10 accessions), whereas 80% of Japan’s accessions were joined into Q3 along with accessions of Mongolia and South Korea (Fig. 2; Fig.S2).

### Genetic diversity of the spinach lines with high vitamin C content

A total of forty spinach genotypes showed high VC content (VC > 1µMol/g FW), of which 31 were obtained from USDA GRIN spinach accessions, while the remaining 9 were from Pop Vriend Seeds. To explore the genetic relationship among the 40 spinach genotypes, a cluster analysis was performed, classifying the genotypes into two major groups, designated as G1 and G2, as depicted in Fig. 3. Group-1 (G1) was comprised of 34 genotypes, which were further divided into four sub-clusters (C1, C2, C3, and C4). The remaining six accessions were clustered in Group 2 (G2). Five of the six accessions in Group 2 were from China and one from Turkey. In Group 1, all Pop Vriend genotypes except one genotype were grouped into cluster 1 (C1). Within this cluster, three genotypes from the United States and one genotype of unknown origin were also clustered. Cluster 2 (C2) had only two genotypes from United States. All genotypes from Turkey (9), except for one, were grouped together in cluster 3 (C3) along with four genotypes from different countries including Afghanistan, Georgia, Greece, and Syria. Cluster 4 (C4) was composed of the remaining seven genotypes, which included four genotypes from China, two from Germany, and one from Pop Vriend Seeds (Fig. 3).

### Association study

**Fig. 4:**
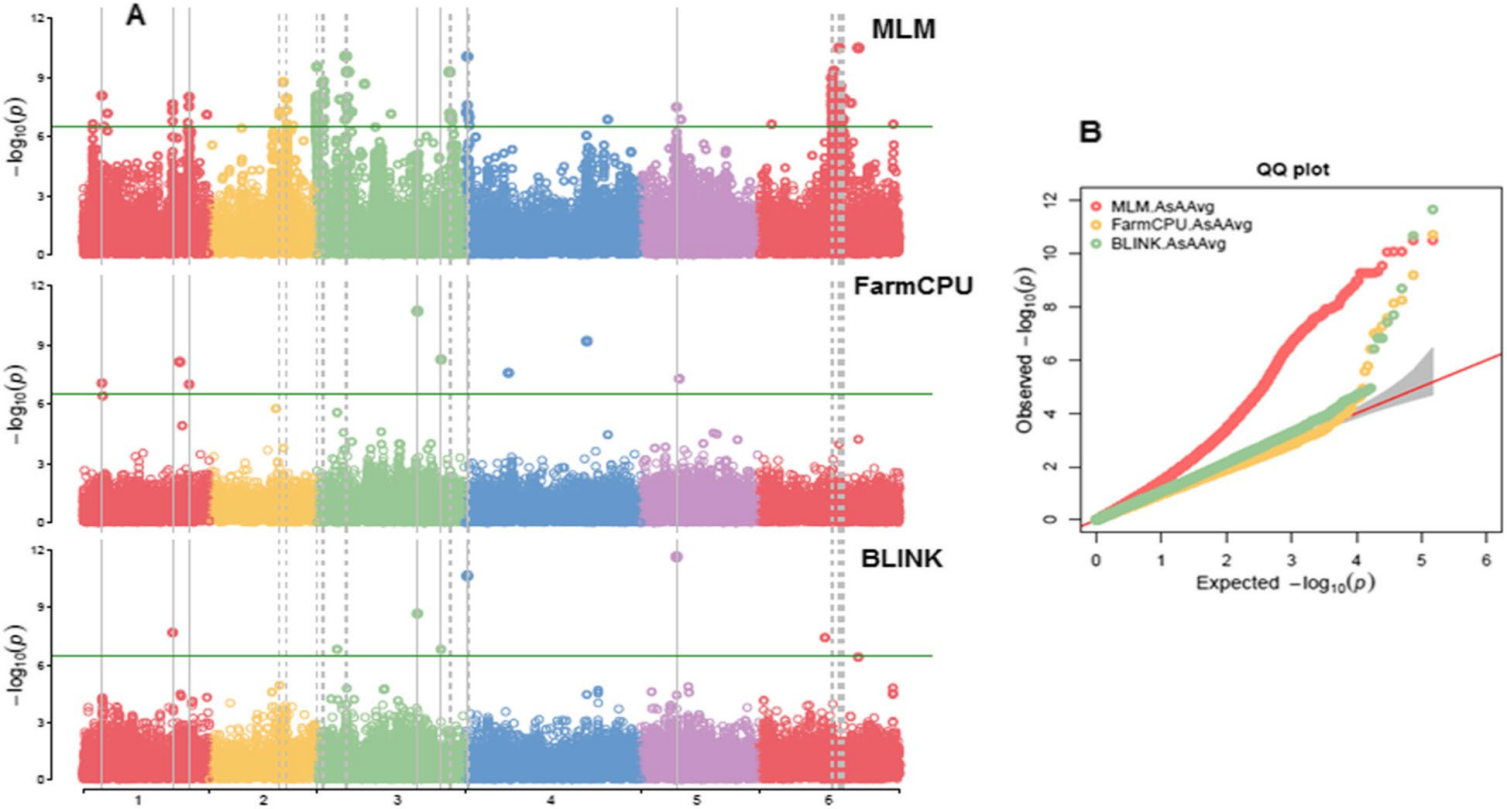
The multiple Manhattan and QQ plots of MLM, FarmCPU and BLINK models for Ascorbic acid (AsA) content by GAPIT 3. **A** In Manhattan plots y-axis indicates log (*P*) value and the x-axis indicate the spinach genome chromosome numbers. **B** In QQ plots y-axis and x-axis denotes observed and expected log (*P*) values

For GWAS analysis, three models, MLM, FarmCPU, and Blink from GAPIT 3, and three models, SMR, GLM, and MLM from TASSEL 5 with PCA = 3 were used to find genetic loci associated to VC content. The observed vs expected LOD [-log_10_(p)] distributions in QQ-plots showed a large divergence from the expected distribution based on multiple QQ plots based on three models (BLINK, FarmCPU, and MLM) in GAPIT 3 and three models (SMR, GLM, and MLM) in TASSEL 5 for the panel of 347 spinach genotypes (Fig. S3; Fig. S4 on right side), indicating there were SNPs associated with VC content in the association panel. The Manhattan plots on the six models (Fig. S3; Fig. S4 on the left side) showed that a dozen SNPs with LOD value greater than 6.47 (significant threshold) on all six chromosomes, indicating that there were SNPs associated with VC content, as shown in Fig. 4 of the multiple Manhattan plot of BLINK, FarmCPU and MLM models in GAPIT 3.

Sixty-two SNPs including 11, 8, 3, 2, 2, and 36 SNPs, located at chromosomes 1, 2, 3, 4, 5, and 6, respectively were associated with the VC content with a LOD [-log_10_(p)] > 6.47 in one or more of these six models (Table S3, Fig. 4, S3, S4). The *t*-test results showed 61 out of 62 SNPs had a LOD > 1.3 (significant difference at *P* = 0.05 level); 58 SNPs had a LOD > 2.0 (significant difference at *P* = 0.01); followed by 54 with LOD > 4.0; 39, LOD > 6.0; 10, LOD > 10.0; and 2, LOD > 30 (Table S3). Most of these SNPs were associated with the VC content at *P* < 0.05, 0.01, 0.0001, and 0.000001 levels, indicating the presence of QTLs for VC content in the respective loci.

Furthermore, by combining the results from the 6 association methods of TASSEL5 and GAPIT3, a total of 12 SNPs significantly associated with VC were identified and distributed among all the 6 chromosomes of spinach genome. Among these SNPs, except SOVchr3_22113096 remaining all of them had LOD > 6.47 in at least two of the 6 methods (Table 2). In addition, in the *t*-test analysis, these SNP markers have shown LOD between 1.6 to 12.26 (significant difference at *P* = 0.05 level) for VC content, indicating that the SNP’s (beneficial alleles) predicted have good association with VC content, and in turn can be used in marker assisted selection.

**Table 2:** SNP markers associated with ascorbic acid (AsA) content are based on six models. The table consists of SNP IDs, chromosomal position, significance values (LOD, t-test) predicted in different methods, beneficial and non-beneficial SNPs respect to VC content

SNP	Chr	Position (bp)	LOD [-log (*P*-value)]							Beneficial_allele	Non_beneficial_allele	Lod > 6.47
			GAPIT 3			Tassel 5			*t*-test			
			Blink	FarmCPU	MLM	SMR	GLM	MLM				
SOVchr1_20183287	1	20183287	0.98	7.06	8.08	10.54	8.31	3.64	4.14	G	A	farmCPU.mlm, smr.glm
SOVchr1_96341316	1	96341316	7.70	0.58	7.64	13.91	8.91	4.88	4.29	T	G	blink,mlm, smr,glm
SOVchr1_114115692	1	114115692	2.10	7.00	8.00	14.49	10.88	2.81	4.33	T	A	farmCPU.mlm, smr,glm
SOVchr2_75097058	2	75097058	3.44	2.78	7.08	12.77	9.54	2.92	6.72	G	T	mlm,t.smr,glm
SOVchr3_22113096	3	22113096	6.83	5.57	4.29	3.86	4.04	3.89	1.85	C	A	blink, (FarmCPU > 5.5; mlm,glm > 4)
SOVchr3_108596086	3	108596086	8.69	10.71	4.61	3.52	2.97	3.01	1.66	G	C	blink.farmCPU
SOVchr3_133911769	3	133911769	6.82	8.25	2.98	1.73	2.74	3.15	1.62	A	G	blink.farmCPU
SOVchr4_2215629	4	2215629	10.66	0.68	10.06	17.03	11.56	4.49	7.31	G	T	blink,mlm smr.glm
SOVchr5_38517805	5	38517805	11.66	0.01	7.49	13.05	8.97	5.04	4.75	A	G	blink,mlm smr.mlm
SOVchr5_41339226	5	41339226	2.43	7.28	4.71	9.61	4.86	3.45	4.62	T	C	farmCPU.smr
SOVchr6_78422108	6	78422108	0.30	0.07	8.98	19.12	19.46	8.88	34.62	T	G	mlm.smr.glm.tmlm
SOVchr6_86008035	6	86008035	1.80	3.99	10.50	15.75	15.81	4.89	12.26	T	C	mlm,smr,glm

Additionally, haplotype block analysis revealed 86 SNPs on 43 different blocks, unevenly distributed on five of six chromosomes in spinach genome (Fig. S5). Chr. 1 has one haplotype block with 2 SNPs, followed by Chr.2 with eleven haplotype blocks, Chr. 3 has twenty three blocks with 50 SNPs, Chr. 4 has one block with 2 SNPs, 24 SNPs distributed on three blocks in Chr. 5 and 8 SNPs on four blocks of Chr.6 (Fig. S5). Our results indicates that all these SNPs on respective haplotype blocks in different chromosomes tend to inherit together. Among the SNPs, SOVchr1_20183287, SOVchr1_96341316 and SOVchr1_114115692 of Chr.1 has a LOD > 7, followed by SOVchr3_22113096, SOVchr3_108596086, and SOVchr3_133911769, of Chr. 3 have LOD > 6.8, SOVchr4_2215629, and SNP of Chr. 4 with LOD > 10, and SOVchr5_38517805, and SOVchr5_41339226 of Chr. 6 with LOD > 7.2 are detected by either or both of the statistically, and computationally powerful GWAS models (BLINK, FarmCPU) (Table 2). The results of this analysis indicate that, upon validation, these SNPs are very strong VC candidate markers for molecular breeding programs aiming to develop VC rich cultivars.

### Candidate Gene Identification/detection

The 62 VC content-associated SNP markers (Table S3), were used for candidate gene prediction as described in methods, resulting in 103 genes flanking with 51 SNPs within the 50 kb region (physical distance) on respective six chromosomes (Table S5). Among them, 35% of the genes are localized in chr. 1, followed by 28.2% of genes on Chr. 6, 11.7% of genes on Chr. 2, and other chromosomes shared with 25.1% of genes. Interestingly 29 genes of Chr. 6 are flanking with 25 SNPs, followed by Chr. 1 and Chr. 2 with 21.6% and 15.7% showing the VC related SNP markers abundance on these chromosomes (Table S5). Of which, 14 genes had one or more associated SNP markers on or within 5 kb distance (Table 3). Most of the genes have been annotated with a wide range of functional categories. Interestingly, three of them are related to Ascorbic acid (= VC) function, including *SOV1g030440* (*Mannose-1-phosphate guanyltransferase*), located at 114,061,560 bp to 114,066,577 bp on chromosome (Chr) 1, *SOV2g020270* (*sucrose-phosphate synthase*), located at 82,610,416 bp and 82,617,190 bp on Chr 2, and *SOV2g023150* (*Ascorbate peroxidase*), located at 89,566,044 bp to 89,569,345 bp on Chr 2, were selected as a candidate genes for VC content in the spinach (Table 3, Table S4).

**Table 3:** Fourteen candidate genes within 5 kb distance and three VC related genes within 20 to 50 Kb region associated with one or more associated SNP markers identified in the GWAS analysis. The table consists of genes and SNPs with respective chromosomal locations and gene functions

Gene	chr	Start_pos (bp)	End_pos (bp)	Gene size (bp)	Annotation_gene_name	SNP	Chr	Pos (bp)	From gene start (bp)	From gene end (bp)	Comment
SOV1g005360	1	26177579	26185874	8295	Transposon, En/Spm-like protein	SOVchr1_26181973	1	26181973	4394	−3901	on gene
SOV1g021120	1	96358503	96366064	7561	V-type ATPase subunit A	SOVchr1_96355403	1	96355403	−3100	−10661	< 3.5 kb
						SOVchr1_96355404	1	96355404	−3099	−10660	< 3.5 kb
SOV1g024720	1	103961184	103966209	5025	NEP-interacting protein%2C putative (DUF239)	SOVchr1_103962443	1	103962443	1259	−3766	on gene
SOV1g030490	1	114112319	114116748	4429	MAR-binding filament-like protein 1 isoform 1	SOVchr1_114115692	1	114115692	3373	−1056	on gene
SOV1g030500	1	114117811	114119588	1777	RING-type domain-containing protein				−2119	−3896	< 2.5 kb
SOV2g017640	2	75018121	75024189	6068	ULP_PROTEASE domain-containing protein	SOVchr2_75014468	2	75014468	−3653	−9721	< 4 kb
SOV2g017650	2	75093004	75093455	451	Inosine-5'-monophosphate dehydrogenase	SOVchr2_75097058	2	75097058	4054	3603	< 4 kb
SOV5g021550	5	38519005	38521418	2413	Embryonic protein DC-8	SOVchr5_38517805	5	38517805	−1200	−3613	< 1.5 kb
SOV5g022430	5	41335623	41336464	841	zf-GRF domain-containing protein	SOVchr5_41339226	5	41339226	3603	2762	< 4 kb
SOV5g022440	5	41336613	41337179	566	Photosystem II protein D1				2613	2047	< 2.5 kb
SOV6g016680	6	70822271	70823847	1576	Expansin	SOVchr6_70820626	6	70820626	−1645	−3221	on gene
SOV6g018170	6	78296537	78303856	7319	Major facilitator superfamily domain-containing protein 12	SOVchr6_78305431	6	78305431	8894	1575	< 2 kb
SOV6g019700	6	86924502	86956934	32432	Bap31 domain-containing protein	SOVchr6_86928259	6	86928259	3757	−28675	on gene
						SOVchr6_86937358	6	86937358	12856	−19576	on gene
						SOVchr6_86937359	6	86937359	12857	−19575	on gene
SOV6g020650	6	91702988	91705430	2442	Solute carrier family 40 protein	SOVchr6_91705644	6	91705644	2656	214	< 0.5 kb
SOV1g030440	1	114061560	114066577	5017	Mannose-1-phosphate guanyltransferase, putative	SOVchr1_114115692	1	114115692	54132	49115	< 50 kb
SOV2g023150	2	89566044	89569345	3301	Ascorbate peroxidase	SOVchr2_89588527	2	89588527	22483	19182	< 20 kb
SOV2g020270	2	82610416	82617190	6774	PREDICTED: probable sucrose-phosphate synthase	SOVchr2_82589739	2	82589739	20677	27451	< 27.5 kb
						SOVchr2_82589758	2	82589758	20658	27432	
						SOVchr2_82590921	2	82590921	19495	26269	< 26.3 kb
						SOVchr2_82590931	2	82590931	19485	26259	

### Genomic prediction of VC content

**Fig. 5:**
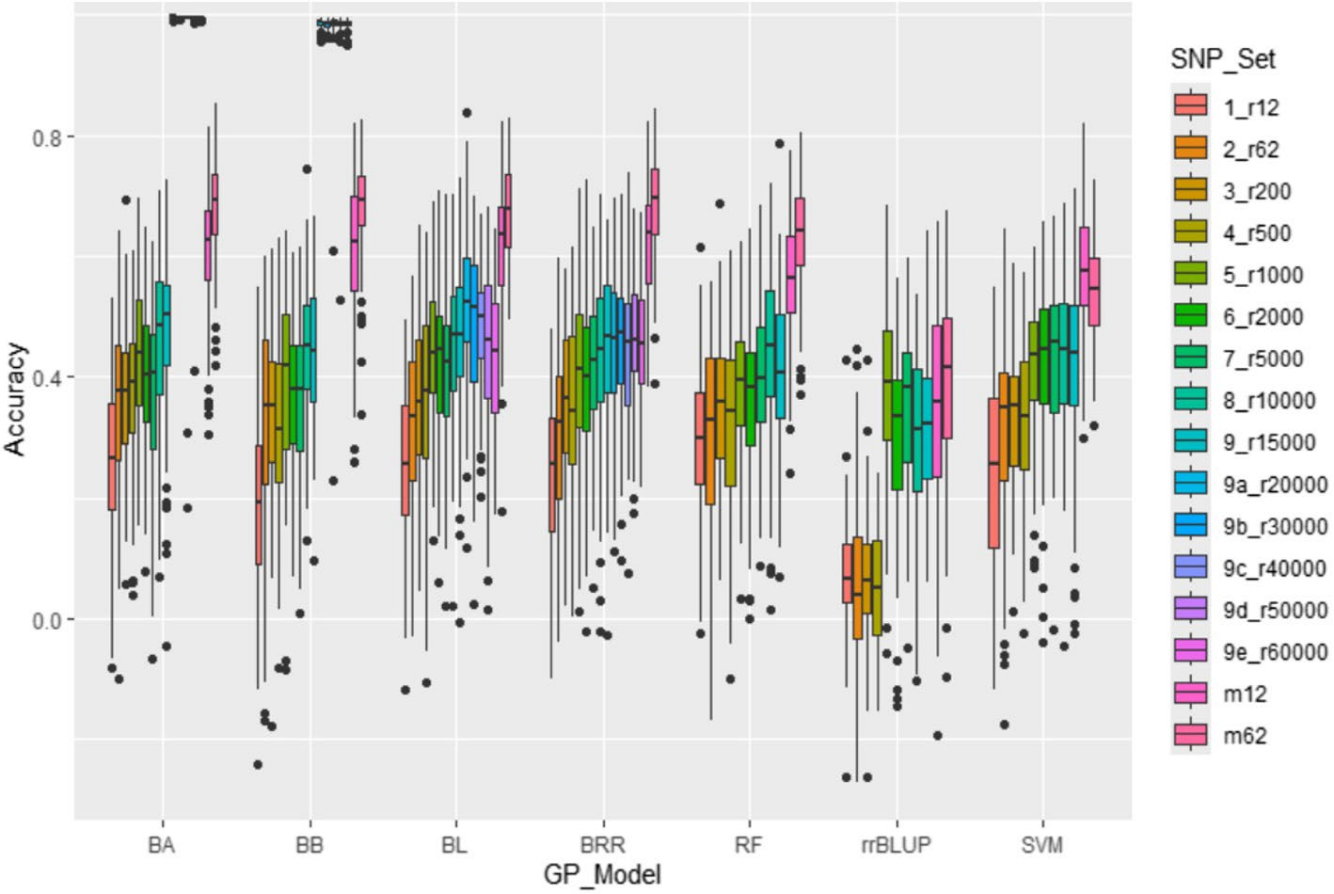
Genomic prediction (GP) (*r*-value) for VC content in 347 spinach lines, estimated by six GP models on ten SNP sets, eight randomly selected SNP sets (1_r12, 2_r12, 3_r200, 4_r500, 5_r1000, 6_r2000, 7_r5000, 8_r10000, 9_ r15000, 9a_r20000, 9b_r30000, 9c_r40000, 9d_r50000, and 9e_ r60000) and two GWAS (m12 and m62) derived SNP marker sets. In the SNP sets, (1_r12) the number followed by r denotes SNP number in each set. The x-axis denotes PA, and y-axis denotes different GP models BA, BB, BL, BRR, RF, rrBLUP, and SVM as abbreviated in materials and methods section

Genomic prediction (GP) for VC was performed using sixteen distinct SNP sets and seven different GP models sets. The SNP sets include randomly selected eight SNP sets, (r12, r62, r200, r500, r1000, r2000, r5000, r10000, r15000, r20000, r30000, r40000, r50000, and r60000) with varying SNPs number per each set, and two GWAS-derived SNP sets with m12 and m62 markers respectively. In total, there were 112 unique combinations for the GP analysis. To obtain accurate results, each GP analysis was run 100 times to calculate GP statistical parameters and correlation coefficient (*r*) values. The average *r*-value (r_Ȳ100_) and standard error (SE) for each GP combination were calculated from the 100 runs (Fig. 5; Fig. S6).

The result of 112 combinations of GP models revealed a downtrend in the average *r*-value (r_Ȳ100_) as the number of randomly selected SNPs < 1000 was reduced as seen in most of the models (Fig. 5; Fig. S6). Specifically, the average *r*-value (r_Ȳ100_) was approximately 0.47 for 10,000 SNPs (r10000) and dropped nearly to 0.26 or less for 12 SNPs (r12) in all the models. However, the average *r*-values (r_Ȳ100_) were greater than 0.4 for those SNP sets that comprised 1000 or more SNPs in most of the models (Fig. 5; Fig. S6A; Fig. S6B). Of seven GP models only BL and BRR has shown PA in the range between 0.4 to 0.6 for even the SNP sets over 1000 to 60,000 (5_r1000 to 9e_r60000), showing that these models are efficient in predicting higher PA even for large SNP sets. Between these two models (BL and BRR), only BRR has shown stable high *r*-value (r_Ȳ100_) over 1000 SNP set (5_r1000) until 60,000 (9e_60000) SNPs and whereas the BL *r*-value declined when using SNPs over 30,000 (6b_r30000) (Fig. S6A; Fig. S6C). Remaining models including BA, BB, RF, rrBLUP and SVM have not shown any *r*-value for SNP sets over 15,000 (9_r15000), which might be due to limitations in either computational or statistical power (Fig. 5; Fig. S6A; Fig. S6B). Moreover, for genomic prediction, a minimum of 1000 randomly selected SNPs required to obtain an average r-value of 0.4 (Fig. S6D).

For GWAS derived SNPs (m12 and m62), except rrBLUP all the models have shown higher average *r*-value (r_Ȳ100_) (Fig. S6). Between these SNP set m12 having approximately *r*-value of 0.5 with RF and SVM and over 0.6 respectively for Bayesian models (BA, BB, BL, and BRR) (Fig. S6 A; Fig. S6B; Fig. S6D). Besides, SNP set m62 having an average approximately 0.6 for RF, and SVM and 0.70 respectively for Bayesian models (Fig. S6 A; Fig. S6B; Fig. S6D). Between randomly selected SNP sets (1 to 9e) and GWAS derived SNP sets (m12, m62), GWAS derived SNP sets have shown high PA with *r*-value. In addition our results have also shown that all the Baysian models given high PA for GWAS derived SNP sets, especially BRR model being the top of them.

## Discussion

### Phenotypic variability

In the present study, the germplasm panel showed a positively skewed distribution for VC content where more than half of the germplasm's accessions exhibited lower values of VC content. Nevertheless, the germplasm displayed sufficient variability for VC content, with a wide range of values (2.086 µMol/g FW) and a high CV of 102.2%. This confirms the germplasm's suitability for GWAS analyses. Twelve accessions with high VC could serve as parental lines in spinach breeding programs to develop new cultivars with elevated VC content. Furthermore, five of these 12 accessions came from the USDA GRIN germplasm, with origins spanning three continents: three from China, one from the United States, and one from Turkey. These accessions could be intercrossed to generate new spinach lines or cultivars containing higher VC content and offering broader environmental adaptability. VC content exhibited a high broad-sense heritability (87.4%) (Table S2), indicating that this trait is highly inheritable, which will, in turn, improve breeding efficiency for VC-enriched spinach varieties.

### Genotypic variability

The genetic relationships among 347 spinach accessions were analyzed through STRUCTURE, PCA and genotype-based phylogenetic analysis. All the analysis revealed the presence of majorly two to three genetic pools (Fig. 3; Fig. S2). Interestingly, the classification (groups and clusters) in our analysis did not correspond to the geographical origins of the spinach accessions. This finding suggests that spinach accessions from diverse regions exhibit genetic similarities, which may be attributed to the exchange of germplasm among different countries or the crossing of gene pools under natural cross events or during spinach breeding programs.

Our study’s clustering pattern, Q1-Q3 (Fig. 3; Fig. S2 and Table. S1) matches the phylogenetic findings of Ribera et al. [67, 68], which analyzed 95 accessions from three spinach species (*S. tetrandra*, *S. turkestanica*, and *S. oleracea*). Phylogeny analysis of both studies show majority of accessions are classified into 2 groups, *S. turkestanica* and *S. oleracea* species in one group, and the other with *S. tetrandra* species, and Asia specific (Eastern and Southern) accessions. Therefore, the findings of both studies confirm that the two gene pools in spinach germplasm can be attributed from *Spinacia tetrandra* Steven ex M. Bieb and *Spinacia turkestanica* Iljin, which are the two closest wild relatives of cultivated spinach. However, due to the insufficient number of wild spinach species in this study, it is not possible to ascertain which group belongs to which gene pool. Nevertheless, the observed clustering patterns are consistent with previous research on spinach germplasm. Population structure analyses conducted on the same germplasm used in this study, as well as on different germplasm, have also revealed two distinct clustering patterns, thus confirming the existence of two gene pools in spinach [6, 25, 50, 67].

The cluster analysis result bolsters our previous recommendation (sec " Phenotypic variability". Phenotypic variability) to select the top five spinach accessions as parents for spinach breeding programs. We find that three of the five accessions belong to Group1, while the remaining two are part of Group 2. This distribution of accessions across different clusters will help breeders to select correct accessions from different clusters rather than from the same cluster in future spinach breeding programs to develop enriched VC spinach cultivars (Fig. 3; Fig. S2 and Table. S1).

The genetic-distance-based phylogenetic analysis of 40 VC-rich spinach accessions revealed a clustering pattern that partially corresponds to their geographical origins. These accessions were grouped into five clusters, categorized into two main groups (G1 and G2). For example, all Turkey spinach accessions except one fell into cluster 3 (C3), while those from the United States and China were mainly in cluster 2 and cluster 5, respectively (Fig. 2). However, a few accessions from these countries were grouped in other clusters as well. Interestingly, this clustering pattern differs from that observed in the broader spinach germplasm, which did not reflect geographical origins. The discrepancy may be attributed to the different number of accessions used for the two analyses. Selection pressure for regional adaptation could explain the observed geographical correspondence in 40 enriched VC content spinach accessions.

Furthermore, it is plausible that seed exchanges among countries during spinach cultivar development could have led to the grouping of some accessions from different countries with Turkey and China, which are dominant in clusters 3, 4, and 5. This indicates that these accessions might share a common genetic ancestry. In addition, the 31 VC content enriched USDA accessions could be used as parents in spinach breeding programs. Finally, the top five spinach accessions with the highest VC content were placed in different clusters, like the cluster analysis of the 347 spinach accessions. This suggests that the genetic distances among these accessions are reliable and could be used as parents in any spinach breeding program aimed at improving vitamin C content.

### SNPs assisted candidate gene identification related to vitamin C

Association analysis was performed with multiple models of GAPIT 3 (MLM, FarmCPU, and Blink) and TASSEL 5 (SMR, GLM, and MLM) software’s for prediction accuracy. Similarly in previous studies, different combination of models were utilized for finding reliable SNPs related to white rust resistance in spinach [30], morphological traits, and resistance to *Peryonella pinodes* in peas [69], agronomic traits such as days to flowering (DTF), plant height (PH), panicle length (PALH), panicle width (PAWD), panicle weight (PAWT), and grain yield (GY) in sorghum [70], justifying our approach. In addition, each model has difference in SNP prediction, the combination of multiple models as performed in this study may reduce false positive associations. In this study 147,977 high quality SNPs predicted through whole genome sequencing, were used in GWAS analysis on a panel of 347 spinach species to predict the SNPs associated with VC. Of these, 62 SNPs were associated to the VC content with a LOD > 6.47 in one or more of these six models (Table S3, Fig. 3; Fig.S3; Fig. S4). The LOD values in each of the 62 SNP markers is not similar across the models, which might be due to the parameters used in each of the models [30, 51, 60, 71–73]. Previously, Rueda et al. [51] predicted that 490 SNP markers associated with VC, and the SNPs were generated based on spinach reference genome Sp75 [50, 54]. In the current study, the SNPs were generated based on the reference Monoe-Viroflay genome annotation [22] (http://spinachbase.org/ftp/genome/Monoe-Viroflay/). Twelve SNPs have been consistent in multiple GAPIT 3, and TASSEL 5 models with high LOD values, indicating that these SNP markers were reliable markers associated with VC content. SNPs with high significance (LOD > 5), SOVchr6_78422108, SOVchr4_2215629, SOVchr6_86008035, SOVchr4_221562 and SOVchr2_75097058 across multiple models may have high association with VC content in spinach. The 12 overlapping SNP markers that were predicted through a combination of models in this study and the 27 SNPs reported by Rueda et al. [51] can increase the probability of identifying genotypes with high and low VC content among diverse germplasm accessions through marker assisted selection. Moreover, the distribution of VC associated SNPs, (50, 24 and 8) on Chr. 3, Chr. 5 and Chr. 6 predicted through GWAS, and LD analysis indicate that these loci may have more candidate genes involved in different biosynthetic pathways related to VC (ascorbic acid) in spinach (Fig. S3; Fig. S4; Fig. S5) [51, 74]. Of the six GWAS models, the SNPs predicted through FarmCPU, BLINK and MLM models has high LOD values, suggesting that these three models will be statistically reliable, and highly efficient for the detection of SNPs related to VC content in spinach germplasm.

A total of 103 genes were identified using the GWAS derived SNPs. Identifying the functions of these genes related to VC will be one of the major objectives for future studies in spinach. Fourteen of these genes are predicted with flanking SNPs either on the gene or within the 5 Kb distance from the gene, these markers will be useful for QTL mapping, and marker-assisted selection related to VC content. Interestingly, three important genes involved in VC biosynthesis, *Mannose-1-phosphate guanyltransferase* (*SOV1g030440*), *Ascorbate peroxidase* (*SOV2g023150*) and *sucrose-phosphate synthase* (*SOV2g020270*) are flanked with one SNP within 50 kb on chr.1, and one and four SNPs within 27.5 kb distance on chr 2 (Table 3). Earlier research studies on these genes reported that, mannose-1-phosphate guanyltransferase enzyme which is encoded by *CVT1* gene is involved in multiple biosynthesis pathways including AsA biosynthesis [75, 76]. *Ascorbate peroxidase* gene is known to play key roles in different molecular networks, including ascorbic acid biosynthesis, and resistance mechanisms related to abiotic stresses in Arabidopsis [77, 78]. *Sucrose-phosphate synthase* which regulates sucrose biosynthesis, has shown to increase VC levels in different crops through sugar metabolisms [79–81]. These 3 genes, are good potential candidates for functional analysis in future studies.

### Genomic prediction for vitamin C content

In this study, GP for VC content was conducted using a five-fold cross-validation approach, with a ratio of 4:1 for training and validation population sets. This approach was applied across the sixteen SNP sets and seven GP models (Fig. 5; Fig. S6). The decision to use a five-fold training and validation sample set was made based on Shi et al. [30], findings. In their study, Shi et al. [30], examined white rust resistance in spinach germplasm using nine different fold sets, ranging from 2 (1:1) to 10 (9:1), for training and validation sets and observed poor performance of larger and smaller training set, with larger training sets exhibiting high standard errors and smaller training sets demonstrating reduced prediction accuracy. Similar findings were reported by Shi et al. [31], and Revelombola et al. [82], on soybean cyst nematode resistance in common bean, and growth habit, flowering time, and grain yield in cowpea. It is plausible that the trend of decreased prediction accuracy in smaller training sets is attributable to the overfitting of the models. Therefore, the chosen methodology balances accuracy and variation for optimal results of the study.

In one hundred and twelve combinations derived from 16 SNP sets and seven GP models, we observed a downward trend in the average *r*-value (r_Ȳ100_) for randomly selected fourteen SNP sets as the SNP number decreased from 60,000 to 12 (Fig. 5; Fig. S6). This trend matches with the findings of Shi et al. [30], who used the same spinach germplasm to study white rust resistance with eight different numbers of SNP sets, ranging from 4836 to 9 SNPs. This observation aligns with several past studies [31, 42, 83, 84], which reported lower PA with fewer SNP markers. However, our results demonstrated that an average *r*-value (r_Ȳ100_) of > 0.4 could be achieved using 1000 or more randomly selected SNPs, implying the feasibility of GS for high VC content in spinach. This finding showed that we need to use more randomly selected SNPs to perform GS for VC selection, since VC content is controlled by more alleles with minor effects than disease-resistant traits like white rust resistance, an average *r*-value (r_Ȳ100_) of > 0.5 was observed with 100 or more SNPs in GP models [30]. The PA in GP could be different due to the phenotypic traits studied, as well as the trait heritability and the number of QTLs controlling the traits [31].

Our analysis also revealed that the two GWAS-associated SNP sets (m12 and m62 SNP sets) exhibited 41% and 48% higher average *r*-values (r_Ȳ100_) compared to the same number of randomly selected SNP sets (Fig. 5; Fig. S6). This observation suggests a potential opportunity to use GWAS-associated SNP markers in GS for enhancing VC content in spinach. This finding is in line with previous studies of Keller et al. [83], Zhang et al. [84] and Shi et al., which also reported increased average *r*-values for GWAS-associated SNP markers compared to random SNPs.

In terms of the performance of seven GP models, we found that, apart from the BRR model, models including BA, BB, RF and SVM exhibited a similar average *r*-value of 0.4 across eleven of sixteen SNP sets. In contrast, BL, and BRR models have shown r-value over 0.45 for all sixteen SNP sets making these models suitable for using on large SNP sets for GP in spinach breeding programs. In addition, BRR model demonstrated a higher PA (*r*-value) for seven randomly SNP sets (r1000 to r60000) compared to other models and *r*-value of 0.6 to 0.7 for GWAS derived SNP sets (m12 and m62) (Fig. 5; Fig. S6). Based on these results, we recommend employing the BRR model for GS in spinach breeding programs targeting high VC content. It is worth noting that the *r*-values for the BA, BB, BL, and BRR models in our study were not similar to those reported by Shi et al. [30], using the same spinach germplasm. Similarly, Shi et al. [31] observed higher *r*-values for the Bayesian models (BA, BB, BL, and BRR) in their research on soybean cyst nematode resistance in common beans. This inconsistency may stem from studying different phenotypic traits in each study as trait heritability and the presence of quantitative trait loci (QTL) are crucial factors that determine the accuracy of GEBVs. The main reason is that the VC content is controlled by more alleles with minor effects in spinach, and our GWAS also showed dozens of QTL regions with SNP markers associated with VC content. Our findings suggest Bayesian model, BRR is highly efficient for GS and in contrary, reports of Shi et al. [30] assertion that the cBLUP model is highly efficient in GS. It is plausible that GP model varies depending on the trait of interest in spinach genomic selection programs.

## Conclusions

In this study, 347 spinach germplasm accessions were tested for vitamin C content under greenhouse conditions. Twelve spinach accessions showed high vitamin C content with over 1.5 µMol/g FW, including 5 USDA accessions and 7 Pop Vriend Seed’s breeding lines, indicating that these lines would be good breeding material to develop high vitamin C cultivars. The genetic diversity analysis showed the 347 genotypes can be divided into 2 clusters or three groups. Genome-wide association study performed in the germplasm set with 147,977 SNPs resulted in 12 SNPs associated with vitamin C, located on all 6 chromosomes of the spinach genome. Seventeen candidate genes have been predicted using vitamin C associated SNPs, of which 14 of them are annotated with a wide range of functions and the remaining 3 genes are related to vitamin C content. Genomic prediction performed with 16 SNP sets using 7 different models resulted in a high *r*-value of 0.7 with the GWAS-derived SNP set (62 vitamin C associated SNPs). The information from this study including the identification of high vitamin C spinach accessions, associated SNP markers, and genomic selection models are great resources for developing new spinach cultivars with high vitamin C content through molecular breeding approaches.

## Supplementary Information

Supplementary Material 1: Table S1. 347 spinach accession /line ID, name, taxonomy, origin, country, cluster, and their ascorbic acid (AsA) content. Table S2. ANOVA for ascorbic acid (AsA) content and Board sense heritability estimation. Table S3. SNP markers associated with ascorbic acid (AsA) content based on six GWAS models and t-test. Table S4. A total of 103 genes located at within 50 kb distance from the associated SNP markers for Ascorbic acid (AsA) content.

Supplementary Material 2: Figure S1. Distribution of the 147,977 single nucleotide polymorphism (SNP) markers on the 6 chromosomes of spinach. Spinach chromosomes are on the vertical axis. Chromosome length in Mb is on the horizontal axis, and the color represents the number of SNPs per 1 Mb window size, SNP density. Figure S2. |Population structure and phylogeny analysis of 347 spinach accessions. A. Population stratification into three population groups based on ΔK analysis. Two (SQ1 and SQ2) and three (SQ1, SQ2 and SQ3) populations estimated from the admixture model (horizontal axis denotes accessions and vertical axis denotes probability values). B. Phylogenetic tree of two and three sub-populations by neighbor-joining (NJ) method drawn by GAPIT 3 in 347 spinach lines, where PCA = 2 (left) and PCA = 3 (right). Figure S3. The Manhattan and quantile–quantile (QQ) plots of BLINK, FarmCPU, and MLM models for ascorbic acid (AsA) content by GAPIT 3. The x-axis and y-axis in the Manhattan plots denote log10 ( *P* ) values and identified SNPs on spinach chromosomes. And x-axis and y-axis in the QQ plots denotes observed and expected log10 ( *P* ) values. Figure S4. The Manhattan and QQ-plot of SMR, GLM, and MLM models for ascorbic acid (AsA) content by TASSEL 5. The x-axis and y-axis in the Manhattan plots denote log10 ( *P* ) values and identified SNPs on spinach chromosomes. And x-axis and y-axis in the QQ plots denotes observed and expected log10 ( *P* ) values. Figure S5. Display of LD plots with SNPs on haplotype blocks in different chromosomes of spinach genome. A. Chr. 1, B. Chr. 2, C. Chr. 3, D. Chr. 4, E. Chr. 5, and F. Chr. 6. The triangle (black color) on respective chromosome represents different blocks with VC related SNP markers. Figure S6. Genomic prediction for ascorbic acid (AsA) content in 347 spinach lines, where (top) fourteen different SNP number sets from 12 randomly selected SNPs to 60,000 SNPs and two GWAS SNP marker sets estimated by seven GP models. A. Genomic prediction of Bayes A (BA), Bayes B (BB), Bayes LASSO (BL), and Bayes ridge regression (BRR); B. Genomic prediction of Random forests (RF), ridge-regression best linear unbiased prediction (rrBLUP) and Support vector machines (SVM). C. Genomic prediction of BRR model. Y-axis shows PA (*r*-value) and X-axis shows GP models in all the plots, and D. Genomic prediction using seven GP models using r1000 and m12 and m62 SNP sets

## Data Availability

The GBS data aligned with the reference genome is available at NCBI with BioProject ID: PRJNA860974. The ascorbic acid of all the accessions used for GWAS analysis is available at Supplementary Table 1. All supplementary materials can be accessed at BMC genomics online.
